# Assessment and cross-validation of calibration transferability between dried blood spot sampling devices for accurate quantification of phosphatidylethanol

**DOI:** 10.1007/s00216-025-05897-x

**Published:** 2025-05-15

**Authors:** J. Hose, M. Juebner, M. Thevis, H. Andresen-Streichert

**Affiliations:** 1 Department of Toxicology, Institute of Legal Medicine, University of Cologne, Faculty of Medicine and University Hospital, Cologne, Germany; 2 Centre for Preventive Doping Research/Institute of Biochemistry, German Sport University Cologne, Cologne, Germany

**Keywords:** Alcohol biomarker, Alternative matrices, Sampling techniques, LC-MS/MS

## Abstract

Phosphatidylethanol (PEth) is a direct alcohol biomarker which is used in a variety of clinical and forensic contexts. Due to its limited stability in whole blood, PEth is routinely analysed using dried blood spots (DBS). There are many different commercially available sampling systems for DBS, but data on their comparability is scarce. The aim of this study was to develop and validate a LC-MS/MS method for the quantification of PEth 16:0/18:1 and PEth 16:0/18:2 for three different DBS sampling systems: Whatman™ filter paper (903 Protein Saver Cards), Mitra^®^ and Capitainer^®^B Vanadate. The transferability of calibration curves and their applicability across these devices should be assessed. The results showed that DBS of all devices could be prepared and analysed using the same procedure. Validation was successful for the devices. The accuracy of QCs was best if quantified using the matching DBS sampling device calibration curve (bias < 15 %). Higher, but still acceptable, bias values were observed when using a Whatman™ calibration curve to quantify PEth in DBS of the Capitainer^®^B Vanadate system and vice versa. Mitra^®^ QCs should only be quantified using device-specific calibration curves, as in all other cases, accuracy exceeded the limit of 15 %. We recommend analysing samples using device-specific calibrations at all times, as determined PEth concentrations might be crucial for patients and should not be subjected to any avoidable uncertainty.

**Graphical Abstract:**

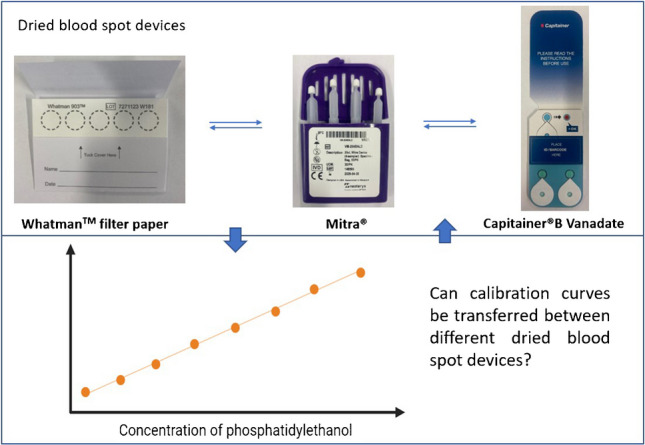

## Introduction

Phosphatidylethanol (PEth) is a group of phospholipids formed by the enzyme phospholipase D (PLD), which catalyses the transphosphatidylation reaction between phosphatidylcholine and ethanol [1–3]. PEth 16:0/18:1 is the most abundant homologue and therefore primarily used in routine analysis and subsequent interpretation. Other PEth species, especially PEth 16:0/18:2, being the second most abundant homologue, are commonly used to verify PEth 16:0/18:1 findings [4, 5].

A major challenge in routine analysis is the reported insufficient stability of PEth in whole blood, unless samples are stored at −80 °C. Therefore, analysis using dried blood spots (DBS) was established as PEth has shown to be stable for several weeks or even months in this form [6, 7]. DBS can be stored at room temperature, thereby simplifying transportation and storage. Frequently used systems are conventional (filter) papers, e.g. Whatman™ filter paper. In addition, volumetric capillary blood collection systems such as Mitra^®^ or Capitainer^®^B have been introduced.

Whatman™ filter papers, such as the 903 Protein Saver Cards, are non-volumetric DBS devices made from cellulose fibres and widely used for a variety of applications, especially due to their cost-effective acquisition. The cards offer space for up to five samples, with a sample size varying from 10 µL up to 80 µL. For quantitative results, samples need to be applied with known and consistent volumes, for example, using a pipette or a Minivette^®^ by Sarstedt™, which requires trained users.

The Mitra^®^ system from Neoteryx^®^ is based on volumetric absorptive microsampling (VAMS^®^) technology. It consists of a plastic handler and a hydrophilic polymer tip, which absorbs blood like a sponge upon contact. Since pipetting is not necessary, this system might be suited for less experienced users; however, care must be taken not to “underload” or “overload” the tip. The product is available with two or four samplers and sample sizes of 10, 20, or 30 µL, but the actual volume is dependent on the manufacturing batch and must be taken into consideration for quantitative results.

Capitainer^®^B sampling systems from Capitainer^®^ are volumetric DBS devices that allow the collection of sample sizes of 10 µL. The blood is added to an inlet and a microfluid channel transfers the sample to a collection disk (Ahlstrom paper, grade 222). For successful sampling, an excess of blood needs to be applied to minimise the risk of incomplete channel filling. Any remaining blood is removed by a dissolving membrane underneath the inlet. Two samples can be collected per card. The Capitainer^®^B Vanadate system was specifically produced for PEth analysis and aims at the prevention of post-sampling PEth formation due to ethanol present in the sample by using sodium metavanadate (NaVO_3_) as a PLD inhibitor [8].

In this paper, an analytical method and validation data for the determination of PEth 16:0/18:1 and PEth 16:0/18:2 using three different frequently used DBS sampling systems (Whatman™ 903 Protein Saver Cards, Mitra^®^ devices, and the Capitainer^®^B Vanadate system) are presented. Furthermore, the comparison of the DBS systems regarding quantitative PEth values was investigated. As laboratories can receive different sampling systems for analysis, it was determined whether each specimen needs to be quantified with a calibration curve using the matching sampling system or whether the calibration curve of another DBS system can be used for accurate results as well.

## Experimental

### Chemicals and reagents

PEth 16:0/18:1 [1.0 mg/mL] (as free phosphate) and PEth 16:0/18:2 [1.0 mg/mL] (as free phosphate) were purchased from Cerilliant (Round Rock, USA). The internal standard PEth 16:0/18:1-D5 [100 µg/mL] (as free phosphate, Cerilliant) was diluted with methanol to yield a working solution of 50 ng/mL. Reference material and working solutions were stored at −80 °C. Methanol and propan-2-ol (both Rotisolv^®^ ≥ 99.95 %, LC-MS-Grade) were purchased from Carl Roth (Karlsruhe, Germany). N-hexane (LiChrosolv^®^, hypergrade for LC-MS) was purchased from Merck (Darmstadt, Germany). Water (HiPerSolv Chromanorm, LC-MS-Grade) was purchased from VWR (Radnor, USA) and formic acid (p.a. ≥ 98 %) from Sigma-Aldrich (St. Louis, USA). An external lyophilised authentic whole blood accuracy control (PEth A 323 WH, target values: PEth 16:0/18:1 = 52.8 ng/mL and PEth 16:0/18:2 = 22.6 ng/mL) was purchased from ACQ Science (Rottenburg-Hailfingen, Germany).

Whatman™ 903 Protein Saver Cards were purchased from Cytiva™ (Marlborough, USA). The 20 µL Mitra^®^ system was purchased from Neoteryx^®^ (by Trajan, Torrance, USA), and the Capitainer^®^B Vanadate system (sample volume: 10 µL) was purchased from Specialty Diagnostix (Passau, Germany).

### Blood samples and preparation of DBS

PEth-negative blood was obtained from six voluntary individuals, who have abstained from alcohol for at least 1 month. Aliquots of the blank blood samples were merged and stocked with PEth 16:0/18:1 and PEth 16:0/18:2 to obtain the required concentrations for the calibration curve and QCs.

DBS on Whatman™ filter paper were created by pipetting 20 µL of the blood samples onto the designated spots of the Saver Cards. The absorptive tip of the Mitra^®^ system was filled with blood by carefully immersing it into the blood samples. DBS using the Capitainer^®^B Vanadate system were created by pipetting an excess amount of blood (20 µL) onto the well of the device. All DBS samples were dried overnight at room temperature and then stored in a mini ziplock bag with silica desiccant until analysis.

### Sample preparation

DBS of Mitra^®^ and Capitainer^®^B systems were removed according to the manufacturer’s instructions and placed in 2-mL Eppendorf tubes. DBS of Whatman™ filter paper were excised in full, folded in half and then placed in 2-mL Eppendorf tubes. Twenty microliters of PEth 16:0/18:1-D5 [50 ng/mL] as an internal standard and 300 µL water/propan-2-ol (30/70 v/v + 0.3 % formic acid) for the extraction were added. After the tubes were closed, they were shaken for 1 h at 1300 rpm and room temperature. One mL n-hexane was added for liquid-liquid extraction, and the tubes were shaken again for 15 min at 1400 rpm. After centrifugation for 10 min at 17,000 g, the supernatant was transferred to a flat-bottom, screw-neck vial. The extract was dried under a nitrogen flow and reconstituted in 100 µL mobile phase A.

### LC-MS/MS

The analyses were carried out on an Agilent (Santa Clara, USA) 1200 Infinity LC^®^ system coupled to a Sciex (Framingham, USA) QTrap^®^ 6500+ for separation and MS/MS detection. The chromatographic method is based on the method published by Aboutara *et al.* [9]. An Acquity UPLC BEH C18 column (2.1 × 150 mm, 1.7 µm; Waters) was operated at 40 °C and a 7.5 min isocratic run with 40 % mobile phase A and 60 % mobile phase B. Mobile phase A consisted of 90 % methanol and 10 % of a 25 mM ammonium acetate solution in water. Mobile phase B consisted entirely of methanol. Ten microliters per sample were injected.

Negative electrospray ionisation was used and specific MS conditions were as follows: curtain gas, 25 psi; collision gas, 6 psi; ion spray voltage, −4000 V; temperature, 500 °C; nebuliser gas: 50; auxiliary gas, 50; declustering potential, −35 V; entrance potential, −10 V; cell exit potential, −20 V. The specific mass transitions and collision energies for each PEth homologue and the internal standard are displayed in Table 1.

**Table 1:** Precursor ions, transition ions and collision energies for each PEth homologue and internal standard used for LC-MS/MS detection. Italicised transition ions were used for quantification

Analyte	Precursor ion (m/z)	Transition ions (m/z)	Collision energy [eV]
PEth 16:0/18:1	701	*281*	−44
		255	−45
		442	−38
PEth 16:0/18:2	699	*279*	−44
		255	−45
		437	−38
PEth 16:0/18:1-D5 (internal standard)	706	*281*	−44
		255	−45
		442	−38

### Validation

The methods for the three DBS sampling systems and two PEth homologues (16:0/18:1 and 16:0/18:2) were validated according to the guidelines of the German Society of Toxicology and Forensic Chemistry (GTFCh) [10, 11]. The Valistat™ 2.0 (Arvecon GmbH, Walldorf, Germany) software was used for evaluation.

#### Selectivity and specificity

Six blank blood samples from different individuals without internal standard and two blank blood samples with internal standard were analysed and examined for any interferences which might influence PEth identification and quantification. In addition, two blank blood samples were spiked with frequently occurring substances and metabolites, especially drugs of abuse, including amphetamine, methamphetamine, MDA, MDEA, MDMA, cocaine, benzoylecgonine, morphine, codeine, dihydrocodeine, buprenorphine, norbuprenorphine, methadone, EDDP, oxycodone, tilidine, nortilidine, tramadol, O-desmethyltramadol, fentanyl, norfentanyl, alprazolam, bromazepam, hydroxybromazepam, diazepam, nordiazepam, oxazepam, flunitrazepam, 7-aminoflunitrazepam, lorazepam, zolpidem phenyl-4-carboxylic acid, THC-COOH, ethyl glucuronide (EtG), naloxone, naltrexone, baclofen and disulfiram. These samples were analysed and examined for interferences as well.

#### Calibration and linearity

An eight-point calibration was prepared by spiking PEth 16:0/18:1 and PEth 16:0/18:2 in whole blood at final concentrations of 10, 25, 50, 100, 200, 300, 400 and 500 ng/mL. All calibration samples were prepared on the same day and then applied to the DBS systems. The calibration curves were analysed on six different days. Analyte areas were plotted against the area of the internal standard.

#### Processed sample stability

Stability of processed samples was verified using low (20 ng/mL) and high (350 ng/mL) concentrations of PEth homologues in pooled whole blood. Six aliquots each were prepared and injected at different times (0, 1, 2, 4, 24 and 48 h after sample preparation). All aliquots were stored in the tray of the autosampler (4 °C) until injection. The absolute peak areas of the analytes were plotted against the time of injection.

#### Limit of detection and quantification

Limits of detection (LOD) and limits of quantification (LOQ) were determined according to the DIN 32645. A six-point calibration of low concentrations with equidistant intervals (2, 4, 6, 8, 10 and 12 ng/mL) was analysed. A significance level of 99 % and a *k*-value of *k* = 3 were used for the software’s calculations.

#### Matrix effects and extraction efficiency

Matrix effects and extraction efficiency were assessed at low (20 ng/mL) and high (350 ng/mL) concentrations of PEth homologues in pooled whole blood. Five standard solutions (in methanol), five spiked blood samples and five PEth-negative blood samples (which were spiked after extraction) at both concentrations were analysed. Matrix effects were determined by plotting the peak area ratios (analyte vs. internal standard) of the spiked extracts against the area ratios of the standard solutions. Extraction efficiency was determined by plotting the peak area ratios (analyte vs. internal standard) of the spiked blood samples against the ratios of the spiked extracts. The acceptance of the guideline is 75–125 % for matrix effects and ≥ 50% for extraction efficiency. Standard deviation was not to exceed 25 %.

#### Precision and accuracy

A homogeneous blood pool of each quality control (QC) at concentrations of 20, 210 and 450 ng/mL for both homologues was prepared and divided into aliquots. Additionally, an external authentic QC (PEth A 323 WH) by ACQ Science (52.8 ng/mL for PEth 16:0/18:1 and 22.6 ng/mL for PEth 16:0/18:2) was aliquoted as well. All QC samples were analysed in duplicate on 8 different days. Repeatability precision and time-different intermediate precision were calculated using the relative standard deviation (RSD). Accuracy was calculated using the bias. Values within ± 15 % for both RSD and bias were considered acceptable according to the guideline.

### Cross-validation of the DBS sampling systems

In addition to the quantification of QCs with the calibrations of the respective sampling systems (see the “Precision and accuracy” section), the QCs were also evaluated using the calibration curves of the other systems. As all three DBS sampling systems use different sample volumes, determined PEth values were adjusted to the actual sample volume of the respective sampling systems. Actual sample volumes were 20 µL for Whatman™ filter paper, 23.7 µL for Mitra^®^ tips and 10 µL for the Capitainer^®^B Vanadate system. Repeatability precision, time-different intermediate precision and accuracy were again calculated using the RSD and bias, respectively.

## Results

### Method

The extraction of PEth 16:0/18:1 and PEth 16:0/18:1 from DBS of Whatman™ 903 Protein Saver Cards, Mitra^®^ and Capitainer^®^B Vanadate was achieved using the sample preparation procedure described above. Using the conditions displayed in Table 1, the LC-MS/MS method could successfully be used for identification and quantification. The Acquity UPLC BEH C18 column proved sufficient for separating the two PEth homologues using a 7.5-min isocratic run.

### Validation

#### Selectivity and specificity

No interferences from the blood matrix, the internal standard or other tested compounds were observed, indicating a sufficient selectivity and specificity of the method for all measured mass transitions and all three DBS sampling systems.

#### Calibration and linearity

The Mandel-F test confirmed linearity (*r* > 0.99), and the Cochran test confirmed variance homogeneity of the calibration from 10 to 500 ng/mL for both PEth homologues and all three DBS sampling systems. No outliers were detected. A weighting of 1/× was advised by the Valistat™ software and therefore applied to all calibrations.

#### Processed sample stability

Processed samples of all sampling systems proved to be stable for up to 48 h after preparation. Absolute analyte areas did not differ more than 15.9 % and 16.4 % between each other for the low and high concentrations, respectively.

#### Limit of detection and quantification

Calibration curves for LOD and LOQ proved to be linear according to the Mandel-F test, and no outliers were detected. Lowest limits for both homologues were achieved using the Capitainer^®^B Vanadate system. A summary of determined limits can be found in Table 2.

**Table 2:** Limits of detection (LOD) and quantification (LOQ) of PEth homologues 16:0/18:1 and 16:0/18:2 for three different DBS sampling systems

Analyte	Whatman^TM^ 903 Protein Saver Cards		Mitra^®^ (VAMS^®^)		Capitainer^®^B Vanadate	
	LOD [ng/mL]	LOQ [ng/mL]	LOD [ng/mL]	LOQ [ng/mL]	LOD [ng/mL]	LOQ [ng/mL]
PEth 16:0/18:1	2.5	6.3	2.8	6.9	1.8	5.2
PEth 16:0/18:2	3.2	6.8	3.5	7.3	2.7	6.5

#### Matrix effects and extraction efficiency

Matrix effects ranged from 92 to 115 % for PEth 16:0/18:1 and from 77 to 106 % for PEth 16:0/18:2. No relevant matrix effects were observed for the Mitra^®^ system, with a mean of 97 % for both homologues at higher concentrations and PEth 16:0/18:1 at the lower concentration (20 ng/mL). For PEth 16:0/18:2 at the lower concentration, ion suppression effects (matrix effect: 77 %) were observed when using the Mitra^®^ system. Whatman™ filter paper and Capitainer^®^B Vanadate systems showed nearly no matrix effects, with means of 103 % and 99 % for both homologues at both concentrations, respectively. Values for the extraction efficiency were relatively low for all homologues and sampling systems, with mean values of 67 %, 57 % and 69 % for Whatman™ filter paper, Mitra^®^ and Capitainer^®^, respectively, however still exceeding the minimum requirement of 50 %.

#### Precision and accuracy

Values for RSD (repeatability precision and time-different intermediate precision) and bias (accuracy) for both PEth homologues and all DBS sampling systems met the criteria of the guideline. Exact RSD values for precision can be taken from Table 3 (for PEth 16:0/18:1) and Table 4 (for PEth 16:0/18:2). Values ranged from 2.2 to 5.5 % for Whatman™ filter paper, 2.4 to 8.4 % for Mitra^®^ and 1.4 to 5.0 % for Capitainer^®^B Vanadate, quantified with the corresponding calibration curves.

**Table 3:**
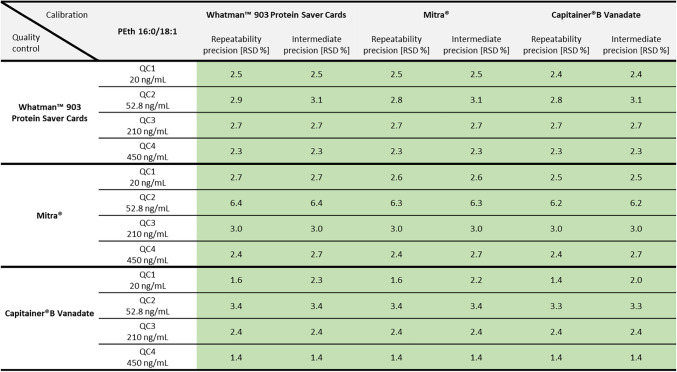
Values for repeatability precision and time-different intermediate precision (using the relative standard deviation (RSD) in %) of the PEth homologue 16:0/18:1 for three different DBS sampling systems. Green-highlighted numbers represent good precision (< 10 %). Yellow areas indicate RSD values between ≥ 10 and ≤ 15 %. Red-highlighted numbers indicate insufficient precision (RSD > 15 %)

**Table 4:**
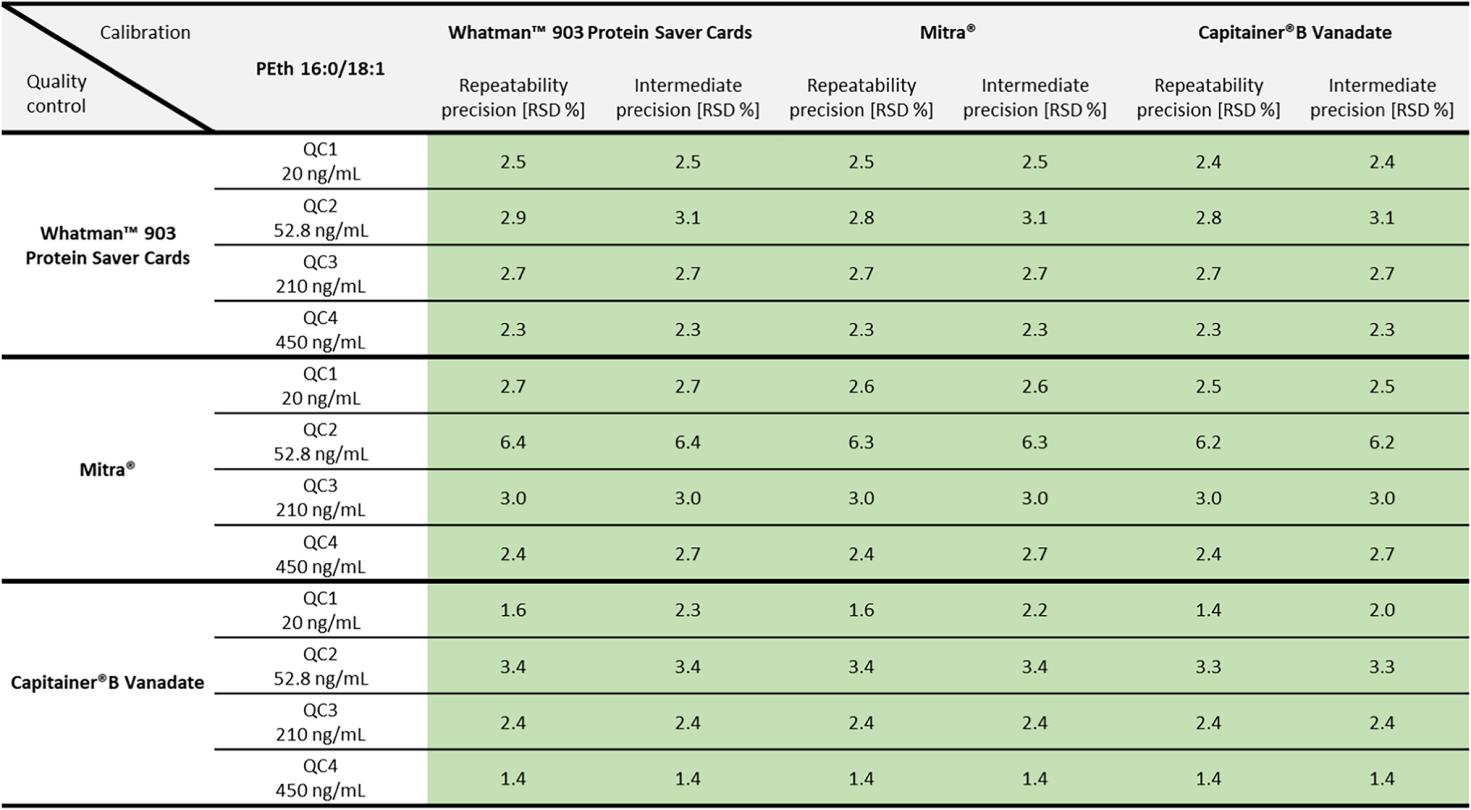
Values for repeatability precision and time-different intermediate precision (using the relative standard deviation (RSD) in %) of the PEth homologue 16:0/18:2 for three different DBS sampling systems. Green-highlighted numbers represent good precision (< 10 %). Yellow areas indicate RSD values between ≥ 10 and ≤ 15 %. Red-highlighted numbers indicate insufficient precision (RSD > 15 %)

Exact bias values for accuracy can be taken from Table 5 (for PEth 16:0/18:1) and Table 6 (for PEth 16:0/18:2). The highest observed bias values were 1.9 % for Whatman™ filter paper, −5.0 % for Mitra^®^ and 1.2 % for Capitainer^®^B Vanadate. In general, the QC2 (external QC, PEth A 323 WH) from ACQ Science showed higher values for precision and accuracy, especially for the Mitra^®^ system, compared to the self-produced ones.

**Table 5:**
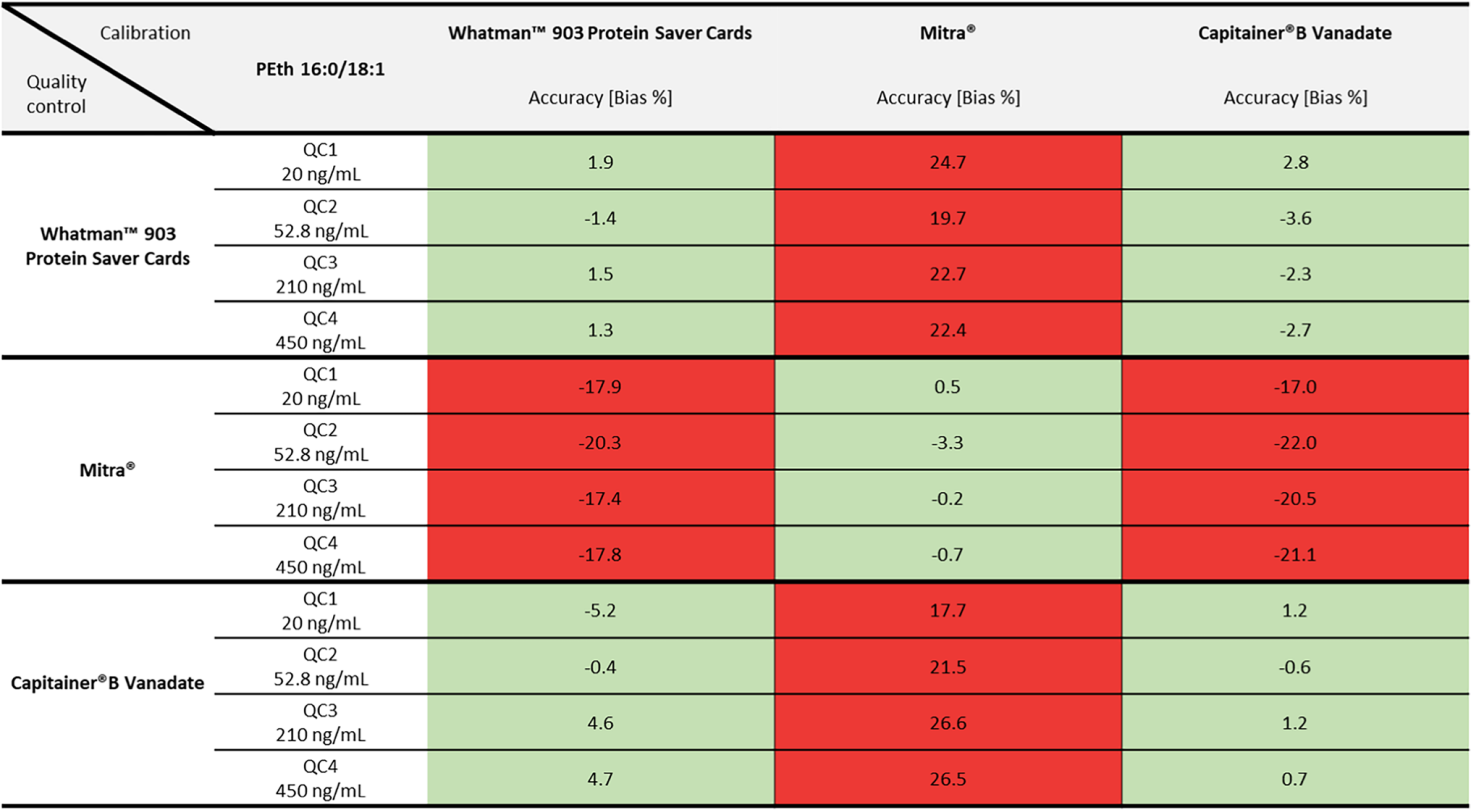
Values for accuracy (using the bias in %) of the PEth homologue 16:0/18:1 for three different DBS sampling systems. Green-highlighted numbers represent good accuracy (< 10 %). Yellow areas indicate bias values between ≥ 10 and ≤ 15 %. Red-highlighted numbers indicate insufficient accuracy (bias > 15 %)

**Table 6:**
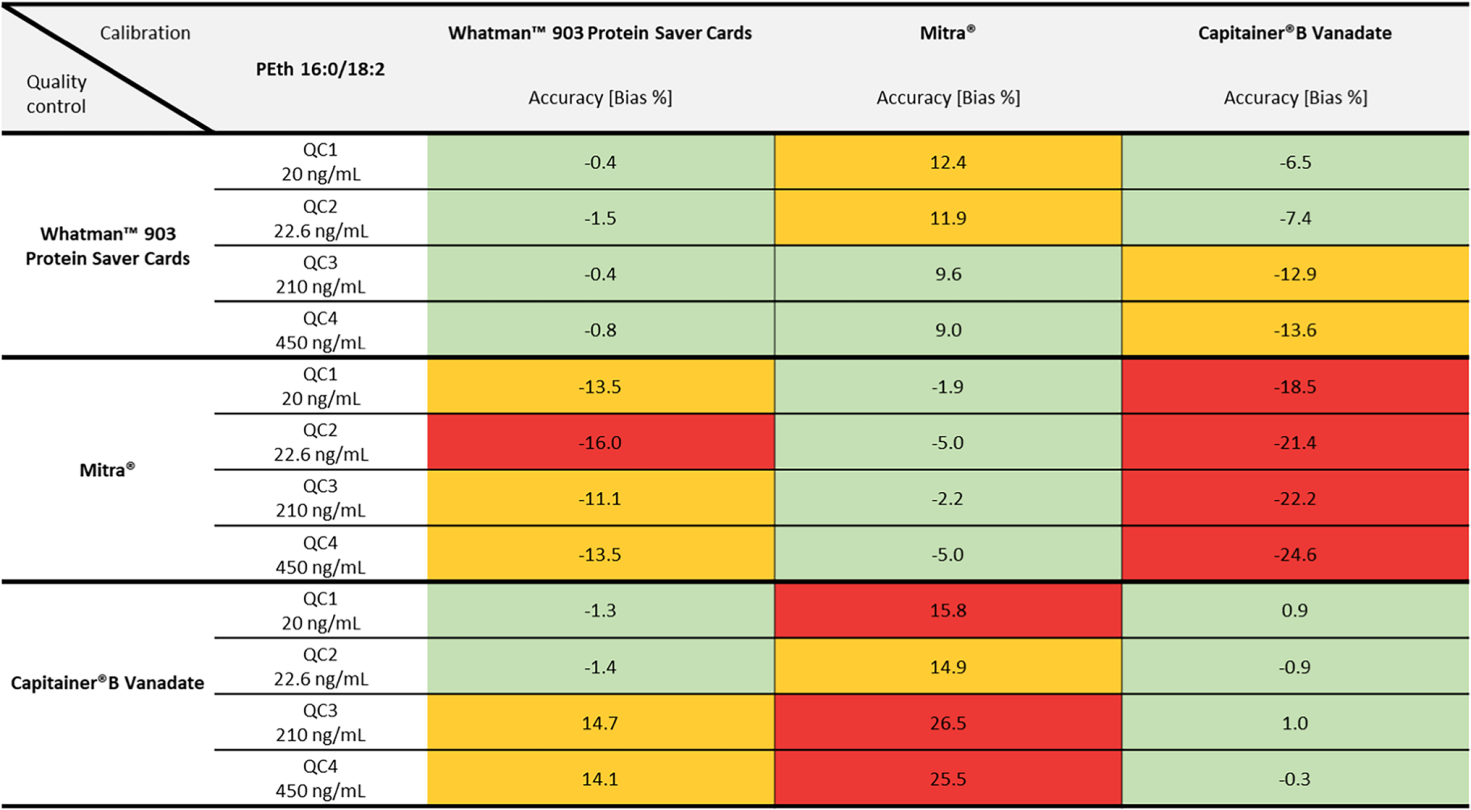
Values for accuracy (using the bias in %) of the PEth homologue 16:0/18:2 for three different DBS sampling systems. Green-highlighted numbers represent good accuracy (< 10 %). Yellow areas indicate bias values between ≥ 10 and ≤ 15 %. Red-highlighted numbers indicate insufficient accuracy (bias > 15 %)

### Cross-validation of the DBS sampling systems

Each quality control was also quantified using the calibration curves of the other DBS sampling systems. The values for precision can also be found in Table 3 (for PEth 16:0/18:1) and Table 4 (for PEth 16:0/18:2). Accuracy values are summarised in Table 5 (for PEth 16:0/18:1) and Table 6 (for PEth 16:0/18:2). Values for RSD and bias for PEth 16:0/18:1 met the criteria of the guideline for QCs of Whatman™ filter paper when quantified with a Capitainer^®^B Vanadate calibration curve and for QCs of Capitainer^®^B Vanadate when quantified with a Whatman™ filter paper calibration curve. The same applies for the PEth 16:0/18:2 homologue; however, higher bias values (> ± 10 % but < ± 15 %) were observed for the QC3 at 210 ng/mL and QC4 at 450 ng/mL. Additionally, for PEth 16:0/18:2, bias values for QCs of Whatman™ filter paper quantified with a Mitra^®^ calibration curve were lower than 15 %. However, the other way around, the bias value of −16.0 % for QC2 exceeds the maximum permitted deviation.

## Discussion

An analytical method for identification and quantification of PEth 16:0/18:1 and PEth 16:0/18:2 was successfully implemented for three different DBS sampling systems (Whatman™ 903 Protein Saver Cards, Mitra^®^ and Capitainer^®^B Vanadate). Sample preparation and LC-MS/MS chromatography could be carried out independently of the sampling system using one method, therefore simplifying the routine laboratory procedure. The smaller calibration range was chosen to ensure sufficient accuracy and thus safety for patients at the low limit value of 20 ng/mL (to distinguish between abstinence and social drinking behaviour) and the value of 200 ng/mL or 210 ng/mL (to distinguish between social drinking behaviour and chronic excessive alcohol consumption). For assessment of abstinence or drinking behaviour, only the homologue 16:0/18:1 is used, but including additional homologues (such as 16:0/18:2) can be helpful to enhance the informative value and aid interpretation [12].

The method was successfully validated for all three DBS sampling systems according to the GTFCh guideline. Selectivity and specificity, linearity and processed sample stability were all satisfactory with regard to the guideline’s specifications [10]. The Mitra^®^ system showed slight ion suppression effects, indicating possible interfering signals from the polymer tip, which have also been reported by Thangavelu et al. [13]. Additionally, the Mitra^®^ system is known for difficulties during the extraction as the analytes might be trapped in the pores of the polymer structure, most likely due to erythrocytes occluding the small openings [13, 14]. This is supported by our findings as the Mitra^®^ system did show the lowest values for extraction efficiency. Interestingly, the Capitainer^®^B Vanadate system did not show any considerable matrix effects even though it contains NaVO_3_ as an inhibitor of the PLD, indicating that this additive does not negatively affect the analysis. The actual concentration of NaVO_3_ contained in the Capitainer^®^ system is not specified by the manufacturer and remains unknown.

Accuracy of QCs was best if quantified using a corresponding DBS sampling device calibration curve. The results indicate a good suitability of the calibration curve of Whatman™ filter paper for quantifying DBS samples of the Capitainer^®^B Vanadate system and vice versa, especially for the PEth 16:0/18:1 homologue. The bias was mostly consistent across all analysed QCs, indicating a possible calibration transferability across the whole calibration range. For the PEth 16:0/18:2 homologue, a good accuracy could only be observed for the low concentrations (20 ng/mL and 22.6 ng/mL). At higher concentrations (210 ng/mL and 450 ng/mL), higher bias values were observed, however still meeting the acceptance criteria of the GTFCh guideline (< 15 %). Mitra^®^ samples should only be quantified using the device-specific calibration curve, as in all other cases, accuracy for PEth 16:0/18:1 values exceeded the accepted limit of 15 %. In comparison, bias values for PEth 16:0/18:2 were lower, some even meeting the acceptance criteria, but still considerably higher with regard to the other DBS sampling devices. Additionally, care must be taken if batches of QCs or samples and calibration curves are not matching, as the actual volume of the Mitra^®^ tips can differ from batch to batch. A correction of determined PEth values might be necessary.

The calibration transferability between Whatman™ filter paper and Capitainer^®^B Vanadate may be attributed to the similar composition of both products. According to the manufacturers, the Whatman™ filter paper and the Ahlstrom paper of the Capitainer^®^ system are both cellulose-based. Therefore, a comparable material texture can be assumed, which might also explain the similar extraction rates. The Mitra^®^ system differs in its composition due to its polymer structure. The extraction efficiency was noticeably lower in this system. Calibration transferability seems more likely when DBS devices exhibit similar material compositions and extraction rates. Laboratories intending to transfer calibration curves between DBS devices should therefore aim for comparable extraction efficiency during method development. This extraction issue might potentially be avoided by integrating the internal standard into the devices, thereby enabling its co-extraction and correction for different extraction efficiencies.

The direction of PEth 16:0/18:1 accuracy (bias) deviations for Mitra^®^ QCs analysed using calibration curves of Whatman™ filter paper or Capitainer^®^ suggests that this homologue may be systematically underestimated under these circumstances. Conversely, when QCs of Whatman™ filter paper and Capitainer^®^ are quantified using a Mitra^®^ calibration curve, there is a risk of systematic overestimation. This is particularly critical for analytes whose interpretation relies on defined threshold values, such as PEth 16:0/18:1. The current scientific consensus is that alcohol abstinence can only be assumed at values below 20 ng/mL [15], but recent studies suggest lowering this cut-off for better detection of low amounts of alcohol consumption [16, 17]. Laboratory methods must therefore prove sufficient accuracy, especially at such low concentrations. It is also advisable to integrate the testing of authentic external QCs to verify the accuracy of developed methods. This helps identify systematic errors, which might arise if only in-house calibration curves and QCs are used.

Any statements made regarding the accuracy apply solely to the method described in this publication. Suitability testing of calibration curves for the quantification of DBS from different sampling devices must be conducted independently in each laboratory and, if need be, should be part of the validation procedure. The transferability of calibration curves between DBS sampling devices for accurate analyte quantification might depend on different factors: the sample size might directly influence the extraction efficiency and matrix effects. When comparing analytes between devices with different sample sizes, a mathematical correction is needed, which might lead to systematic errors. Extraction, matrix effects, and the drying process can also be dependent on the absorbent material of DBS devices. The homogeneity of analytes on each device (e.g. “volcano effect” [18]) might also differ from material to material and must always be considered if sub-punches of spots are used for analysis. As PEth is formed and located at the surface of erythrocytes, the amount of PEth is dependent on the haematocrit [1, 19]. For devices with fixed blood volumes, such as Mitra^®^ and Capitainer^®^B, this haematocrit effect is described as negligible [20, 21]. However, it remains to be discussed whether this also applies to PEth, as its concentration fundamentally depends on the number of erythrocytes within the DBS sample. Carling et al. [14] did postulate that the haematocrit of blood samples can indeed affect extraction and recovery of Mitra^®^ systems as the amount of erythrocytes might influence the occluding of the pores in the polymer material. For Whatman™ filter paper, a potential bias must always be taken into consideration when the DBS is not used as a whole but only a partial punch is used for analysis.

## Conclusion

The described method for identification and quantification of PEth was successfully developed and validated for three different DBS sampling systems (Whatman™ 903 Protein Saver Cards, Mitra^®^ devices with VAMS^®^ technology and the Capitainer^®^B Vanadate system). Sample preparation and LC-MS/MS analysis could be carried out independent of the device, thereby simplifying the laboratory procedures. Even though our study suggests that, using this method, QCs of Whatman™ filter paper (903 Protein Saver Cards) can be quantified using a calibration curve applied to the Capitainer^®^B Vanadate system and vice versa, best accuracy was still achieved when using corresponding calibration curves. For the Mitra^®^ system, sufficient accuracy of QCs was only achieved when using a Mitra^®^ calibration curve. The transferability of calibration curves is dependent on the used DBS sampling devices, which each have their own specific properties and characteristics. Each might influence the sample preparation, extraction efficiency and analysis and therefore adds uncertainty to the method. As the interpretation of PEth results is based on cut-off values, we recommend using the corresponding calibration curve at all times.

## Data Availability

Data can be provided upon reasonable request to the corresponding author.
